# A case report: interstitial pneumonia following treatment of gastric cancer with sintilimab in combination with S-1

**DOI:** 10.3389/fphar.2025.1508558

**Published:** 2025-02-05

**Authors:** Pei Zhu, Qingming Sun, Sheng Xu, Wanhui Dong

**Affiliations:** ^1^ Department of Medical Oncology, Anhui University of Traditional Chinese Medicine Affiliated Liu’an Traditional Chinese Medicine Hospital, Lu’An, China; ^2^ Department of Medical Oncology, Lu’an Hospital of Traditional Chinese Medicine, Lu’An, China

**Keywords:** chemotherapy, case paper, immunotherapy, interstitial pneumonia, S-1, sintilimab

## Abstract

**Background:**

Interstitial pneumonia is a group of pathologies affecting the pulmonary interstitium, characterized by interstitial fibrosis and extensive alveolar consolidation. This disease can extend to the surrounding blood vessels and pulmonary interstitium, sometimes affecting the entire lung, resulting in functional limitations, including restrictive ventilatory defect, impaired gas exchange, and hypoxemia. Severe interstitial pneumonia can lead to death. Antitumor drugs can induce interstitial pneumonia. Sintilimab is an immune checkpoint inhibitor, a recombinant fully human immunoglobulin G-type programmed death protein-1 monoclonal antibody inhibitor. S-1 is a compound preparation consisting of gimeracil, oteracil potassium, and ftorafur. There have been cases of interstitial pneumonia caused by treatment with sintilimab or S-1 in clinical settings, but no cases of interstitial pneumonia caused by treatment with a combination of sintilimab and S-1 have been reported.

**Case report:**

A patient diagnosed with gastric cancer underwent nine courses of treatment using a chemotherapy regimen of combined oxaliplatin S-1., Due to severe bone marrow suppression and gastrointestinal adverse reactions, the treatment was switched to sintilimab in combination with S-1therapy., This change resulted in the development of interstitial pneumonia, as revealed by non-contrast chest Computed Tomography scans. Following a review of blood test results and a multidisciplinary consultation, we suspect that the interstitial pneumonia may have been caused either by Sintilimab alone or by the combined effects of sintilimab and S-1. The treatment was discontinued, and after receiving adequate glucocorticoid therapy, the pulmonary lesions showed slight improvement.

**Conclusion:**

This case provides a clinical reference, indicating that prior touse of sintilimab in combination with S-1 antitumor regimen, a comprehensive baseline assessment should be conducted, including blood routine examination, enzyme tests, and pulmonary imaging examination, with close monitoring of the patient’s pulmonary condition. If drug-induced lung injury is suspected, the medication should be discontinued immediately, and appropriate treatment should be initiated promptly.

## Introduction

Interstitial pneumonia (IP) is a pulmonary interstitial inflammation and fibrotic disease caused by various etiologies. It primarily affects the alveolar cavities and pulmonary interstitium, including alveolar epithelial cells, capillary endothelial cells, basement membranes, and perivascular and perilymphatic tissues. This condition ultimately leads to pulmonary interstitial fibrosis (Wang et al., 2023). The administration of antitumor drugs can lead to the occurrence of IP (Mert et al., 2020). Sintilimab, an immune checkpoint inhibitor, enhances the immune system’s attack on tumor cells by blocking the binding of Programmed Death-1(PD-1) to its ligands. There have been clinical cases of interstitial pneumonia occurring following treatment with PD-1 inhibitors (Chen et al., 2024). S-1 is a compound antitumor medication consisting of gimeracil, oteracil potassium, and ftorafur. Clinically, cases have been reported where administration of S-1 has led to acute IP (Li et al., 2014; Xu et al., 2024). We report a clinical case of extensive IP in the lungs of a gastric cancer patient following treatment with the combination of sintilimab and S-1. This case serves as a reference for potential occurrences of IP during the clinical application of this combined treatment regimen of sintilimab and S-1.

## Case presentation

A patient presented with symptoms of hematemesis and melena without an apparent cause in February 2022. One month later, a gastroscopy with biopsy was performed at the Department of Gastroenterology, Lu’an People’s Hospital, revealing a poorly differentiated adenocarcinoma at the junction of the gastric antrum and body. A week later, the patient underwent a palliative total gastrectomy with esophageal-jejunal Y-anastomosis at Anhui Provincial Hospital. During the operation, multiple metastatic lymph nodes were found in the mesenteric peritoneum. The tumor was located at the vertical part of the lesser curvature of the gastric body, measuring approximately 3*4 cm, and presented as an ulcerative mass with invasion. Postoperative pathology, revealed: Poorly differentiated adenocarcinoma with ulceration on the lesser curvature of the gastric body, measuring 3 cm*1.5 cm*1 cm, with invasive growth into the subserosal adipose tissue, no vascular thrombi, and nerve invasion noted. No cancer involvement was detected at the two anastomotic sites examined. No lymph nodes were detected on the greater curvature of the stomach (−) 0/12, and on the lesser curvature (−) 0/7. Immunohistochemical results (IHC22-04212) of the cancerous tissue showed: HER-2 (−), MLH1 (+), MSH2(+), MSH6(+), PMS2(+). The patient has never smoked, does not have chronic bronchitis or chronic obstructive pulmonary disease, and has no family history of lung or gastric cancer. The patient was diagnosed with gastric cancer following surgery, and the pathological stage was classified as pT4aN0M1, indicating an advanced stage (Stage IV). According to the Karnofsky Performance Status (KPS), the patient’s score was 80 points.

**FIGURE 1:**
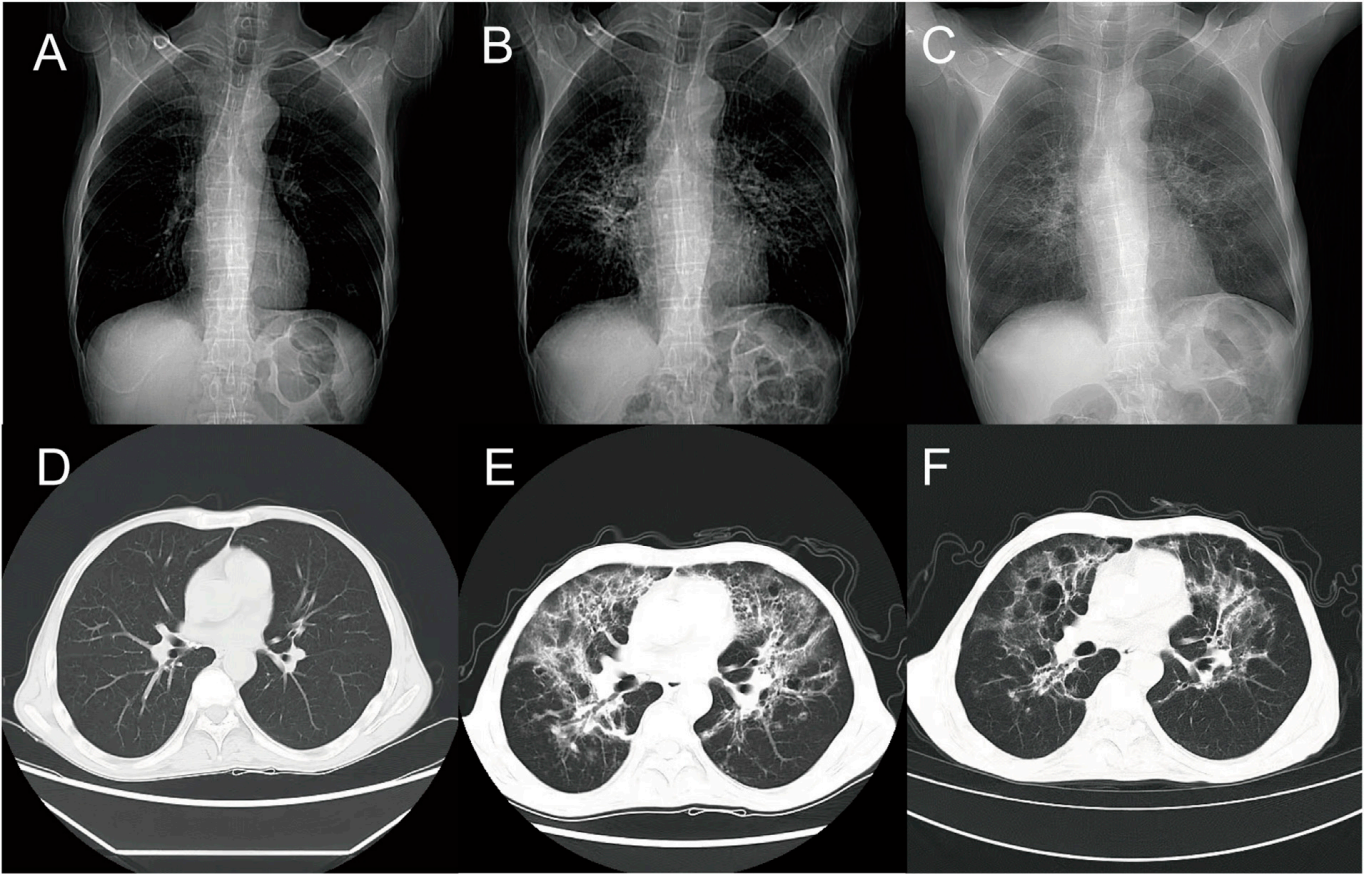
Chest CT. **(A, D)** After nine courses of combined treatment with oxaliplatin and capecitabine, the lung markings became clear, with minor visible streak-like and faint patchy high-density opacities. **(B, E)** Following one course of combined treatment with sintilimab and tegafur, the lung density decreased, revealing multiple streak-like, ground-glass, and patchy high-density opacities with ill-defined borders and uneven density, presenting a grid-like and cast-like change. **(C, F)** After discontinuation of antitumor drugs and administration of hormonal therapy, the lung density further decreased, showing multiple streak-like, ground-glass, and patchy high-density opacities with ill-defined borders and uneven density, presenting a grid-like and cast-like change.

The patient underwent monthly chemotherapy sessions at the Oncology Department of Lu’an Traditional Chinese Medicine Hospital from April 2022 to November 2022. The treatment protocol involved oxaliplatin 150 mg administered via intravenous drip on day 1 (manufactured by Jiangsu Hengrui Medicine Co., Ltd., national drug approval number H20213312, 10 mL: 50 mg/bottle) and capecitabine 50 mg orally, bid, on days 1–14 (manufactured by Jiangsu Hengrui Medicine Co., Ltd., national drug approval number H20113281, 25 mg × 28 capsules). Nine chemotherapy sessions were conducted. One month later, the patient was hospitalized again for a non-contrast chest Computed Tomography (CT) scan, and the results indicated that there were no significant lung lesions (Figures 1A, D).

Due to significant bone marrow suppression and gastrointestinal reactions during chemotherapy, the patient was switched to the “Sintilimab 200 mg iv d1 (Innovent Biologics, Suzhou, China, National Medical Product Approval Number: S2018001, 100 mg (10 mL)/bottle) and S-1 40 mg po bid d1-14”regimen. One month later, the patient exhibited symptoms of fever, cough, sputum production, and chest tightness. Upon admission, the patient’s chest non-contrast CT scan revealed bilateral emphysema, pulmonary inflammatory lesions, and predominantly interstitial lesions (Figures 1B, E). The patient’s KPS score was 50 points. Subsequently, the patients underwent examinations for a complete blood count, procalcitonin levels, C-reactive protein levels, sputum culture, and blood culture. The results indicated that the white blood cell count was 7.07 × 10^9/L, neutrophil count was 6.03 × 10^9/L, procalcitonin was 0.15 ng/mL, and C-reactive protein was 104.80 mg/L. Due to significant shortness of breath following physical activity, the patient declined the bronchoalveolar lavage examination. Following a multidisciplinary consultation, the possibility of severe interstitial lung injury caused by the combination therapy of sintilimab and S-1 could not be ruled out, leading to the discontinuation of this anti-tumor regimen. The patient received continuous administration of methylprednisolone sodium succinate (80 mg iv qd) for seven consecutive days. Upon re-examination, the blood test results revealed the following: white blood cell count was 9.70 × 10^9/L, neutrophil count was 8.88 × 10^9/L, procalcitonin level was 0.08 ng/mL, and CRP level was 3.47 mg/L. The patient continued to receive ongoing treatment with methylprednisolone sodium succinate (40 mg iv qd) for 6 days, followed by a reduced dosage of methylprednisolone sodium succinate (20 mg iv qd) for an additional 6 days. Subsequently, the patient was administered methylprednisolone sodium succinate (10 mg iv qd) for 3 days. As a result of this treatment regimen, the patient’s respiratory symptoms improved, and the patient’s KPS score increased to 60 points. A follow-up examination revealed a reduction in the exudation of lung lesions; however, there was no significant change in the area (Figures 1C, F). After discharge, the patient was prescribed methylprednisolone tablets at a dosage of 4 mg orally once daily for 1 month, after which the treatment was discontinued. The doctors were generally satisfied with the treatment outcomes regarding the adverse reactions caused by sintilimab in combination with S-1. However, the patients expressed dissatisfaction due to a decline in their physical condition and prolonged periods of bed rest. After 2 weeks, the patient was contacted for a follow-up via telephone. The patient reported experiencing chest tightness following physical activity, but no other new symptoms of discomfort were noted.

## Discussion

IP is a collective term for a group of diffuse pulmonary diseases primarily affecting the interstitium and alveolar cavities, leading to the loss of alveolar-capillary functional units (Interstitial Lung Disease Subspecialty Group R M B and Chinese Medical Association, 2016). IP may exacerbate pulmonary injuries and pose life-threatening risks to patients, making early clinical identification and timely treatment of critical importance (Xu et al., 2024). There have been clinical reports on a case of IP induced by treatment with sintilimab in a patient with Hodgkin’s lymphoma (Liu and Luo, 2023). There are also clinical cases of severe drug-induced interstitial lung disease reported to be caused by S-1 (Yang et al., 2018). Currently, there are no clinical reports on extensive IP resulting from the combined treatment of sintilimab and S-1.

IP is a pulmonary inflammatory and fibrotic disease caused by various factors, including allergies, medications, inorganic mineral dust, and connective tissue diseases. If left untreated, it can lead to respiratory failure and even death (Shi and Zhang, 2023; Ma and Zhang, 2023). Early diagnosis of IP is crucial, yet it remains challenging to make an accurate diagnosis and differentiate it from other conditions (Hu, 2015). The early clinical manifestations of IP are nonspecific, often presenting as fever, cough, expectoration, and progressive dyspnea (Liu and Wu, 2024; Yu et al., 2020). The etiology of IP is diverse (Shao et al., 2024; Liang et al., 2022; Yang and Shen, 2020; Jin and Chen, 2019). Compared to conventional CT, high-resolution CT (HRCT) has higher resolution, offering better diagnostic efficacy for interstitial pneumonia (IP) and preventing misdiagnosis (Chen and Wu, 2024; Zhu et al., 2023). Common HRCT findings of IP include ground-glass opacities, excessive linear opacities between and within pulmonary lobules, thickening of interlobular septa, increased interlobular parenchyma thickness, bronchiectasis, honeycombing, and emphysema (Li et al., 2022; Shao et al., 2024). Pathological diagnosis can be achieved through bronchoalveolar lavage, transbronchial lung biopsy (TBLB) and transbronchial lung cryobiopsy (TBLC) (Pu et al., 2023; Zhao et al., 2022; Wang and Feng, 2022; Lin et al., 2019). Clinically, glucocorticoids are therapeutic agents for interstitial lung diseases (Liu et al., 2021; Xiong and Lu, 2016).

After undergoing treatment with sintilimab in combination with S-1, the patient exhibited clinical symptoms, including fever, cough, expectoration, and chest tightness. Based on these clinical manifestations, a pulmonary infection or drug-induced pulmonary adverse reactions were considered. Blood tests were conducted, revealing negative results for white blood cells, neutrophils, procalcitonin, blood culture, and sputum culture, with only the CRP indicator returning a positive result. Bronchoalveolar lavage, TBLB, and TBLC are of significant value in diagnosing and differentiating interstitial lung diseases. However, due to the patient’s poor physical condition, they refused these examinations. After a multidisciplinary consultation, a pulmonary infection-induced IP was ruled out, and it was concluded that the IP was caused by the combination therapy of sintilimab and S-1. There are two potential scenarios that can lead to IP: one is caused by sintilimab alone, while the other results from the combination therapy of sintilimab and S-1. The pathogenic mechanisms of these two drugs differ. As an immune checkpoint inhibitor, the sintilimab antibody restores the proliferation and effector capabilities of T cells by targeting the interaction between the PD-1 protein and its ligand, thereby blocking the immune escape pathways of tumor cells and ultimately killing them. However, the activated immune system may also lead to autoimmune lung injury in normal tissues (Baik et al., 2021; Zhang et al., 2020; Zhu et al., 2023; Li et al., 2022). After conversion to 5-FU, S-1 inhibits DNA synthesis, inducing cell death and potentially triggering apoptosis in lung epithelial and endothelial cells. This process releases the inflammatory mediator TGF-β, which promotes fibroblast proliferation and participates in the pulmonary fibrosis process (Hou et al., 2015; Pu et al., 2023). The patient should undergo a comprehensive evaluation, which includes pulmonary function tests, blood gas analysis, multiple sputum bacterial cultures, HRCT scans, bronchoscopy, and lung tissue biopsy, depending on the patient’s specific condition. The patient discontinued the combined therapy of sintilimab and S-1 and was subsequently administered an adequate dosage and duration of glucocorticoid therapy, which resulted in an improvement in the patient’s condition.

In this case, the patient developed a fever and gastrointestinal symptoms following treatment with sindelimab in combination with S-1. A multidisciplinary consultation was conducted based on the results of blood tests and imaging studies to rule out pulmonary infection. The symptoms may have been caused by sindelimab or IP resulting from the combination of sindelimab and S-1. After treatment with glucocorticoids, the patient’s condition improved slightly. IP is a potentially life-threatening complication that requires careful management. Clinically, it is essential to prevent patients from developing IP as a result of PD-1 inhibitors. First, for patients receiving anti-tumor treatment with PD-1 inhibitors for the first time, a comprehensive baseline assessment should be performed. This assessment should include a complete blood count, enzyme tests, and pulmonary imaging. Such evaluations are crucial for monitoring disease changes during the immunotherapy process, enabling timely and appropriate interventions. We should identify patients at risk. The following conditions are classified as high-risk: a history of pulmonary diseases, including chronic bronchitis and COPD, as well as previous abnormal pulmonary imaging findings such as radiation-induced pneumonia and pulmonary fibrosis. Additionally, patients who have undergone multiple courses of PD-1 inhibitor therapy, as well as those receiving combined PD-1 inhibitor therapy, are also considered high-risk. Multidisciplinary team (MDT) consultations should be conducted to facilitate early diagnosis and timely treatment, prevent disease progression and treatment delays, and avoid irreversible damage to patients.

## Conclusion

This case underscores the importance of conducting comprehensive baseline assessments and closely monitoring pulmonary conditions in patients undergoing treatment with the combination of sintilimab and S-1 in clinical practice to prevent the occurrence of IP. Once IP is diagnosed, the relevant medications should be immediately discontinued, and appropriate treatment measures initiated to prevent serious adverse events and ensure the safety of the patient’s life.

## Data Availability

The original contributions presented in the study are included in the article/supplementary material, further inquiries can be directed to the corresponding author.
